# Particle and Carbon Dioxide Concentration Levels in a Surgical Room Conditioned with a Window/Wall Air-Conditioning System

**DOI:** 10.3390/ijerph17041180

**Published:** 2020-02-13

**Authors:** Marcelo Pereira, Arlindo Tribess, Giorgio Buonanno, Luca Stabile, Mauro Scungio, Ilaria Baffo

**Affiliations:** 1 Department of Refrigeration and Air Conditioning, Federal Institute of Education, Science and Technology of Santa Catarina, São José 88103-310, Brazil; marceloluizpereira@yahoo.com.br; 2 Department of Mechanical Engineering, University of São Paulo, São Paulo 05508-030, Brazil; atribess@usp.br; 3 University of Cassino and Southern Lazio, 03043 Cassino, Italy; l.stabile@unicas.it; 4 School of Engineering, University of Tuscia, 01100 Viterbo, Italy; mauro.scungio@unitus.it (M.S.); ilaria.baffo@unitus.it (I.B.)

**Keywords:** surgical room, air conditioning systems, particle number concentration, CO_2_ concentration, air contamination

## Abstract

One of the most important functions of air conditioning systems in operating rooms is to protect occupants against pathogenic agents transported by air. This protection is done by simultaneously controlling the air distribution, temperature, humidity, filtration and infiltration from other areas etc. Due to their low price, simple installation, operation and maintenance, window/wall air conditioning system have largely been used in operating rooms in Brazil, even if these types of equipment only recirculate the air inside the room without appropriate filtration and renovation with outdoor air. In this context, this work aims to analyse the performance of the window/wall air conditioning systems on indoor air ventilation in operating rooms by measuring particle number concentrations and carbon dioxide concentrations during different surgical procedures, in a single surgical room and in the nearby areas (corridor) for two cases: single surgery and two subsequent surgeries. In addition, the efficiency of the analysed air conditioning system was evaluated by comparing the ventilation level calculated in the surgical room with the ventilation required in order to maintain the carbon dioxide concentration within acceptable levels. The results showed that this type of air conditioning system is not appropriate for use in operating rooms since it cannot provide an adequate level of ventilation. The CO_2_ concentrations during surgeries, in fact, significantly exceeded acceptable values and a simultaneous increase in particle number concentration was observed. The results also showed that there is a high risk of contamination between subsequent surgeries in the same surgical room, due to residues of contaminants transported by the particles emitted during the surgeries that were not removed from the operating room by the air conditioning system. The particle number concentration measured in the second surgery, in fact, was approximately six times higher than in the first surgery.

## 1. Introduction

In an operating room the air conditioning system should guarantee precise control of environmental conditions in order to dilute and remove contaminants that may be in the form of odour, viruses, microorganisms, hazardous chemicals, radioactive substances etc. [1,2,3]. In these rooms, contaminant agents such as biological (microorganisms-fungi, bacteria and viruses), chemical (waste anaesthetic gases, carbon dioxide, etc.) and particulate matter can easily be dispersed by draughts or can remain in suspension in the air for several hours [4,5,6,7]. Additionally, depending on their size, airborne infectious particles in the operating rooms can be broadly classified as follows: bacterial cells and spores (from 0.3 to 10 μm in diameter); fungal spores (from 2.0 to 5.0 μm); viruses (from 0.02 to 0.30 μm in diameter) [8,9,10]. Generally, these agents have three origins: i) generated inside the operating room, ii) infiltrated from adjacent areas and iii) introduced into the room via the ventilation system from outdoors.

Air conditioning systems used inappropriately, besides endangering the health and well-being of the occupants of an operating room, cause the microorganisms carried by droplets or dust particles to sediment on surgical wounds, or on instruments and materials, such as gloves, gauze and clothing. That is, these microorganisms can subsequently enter the surgical wounds of the patients, by indirect transfer or direct deposition into the wound itself. In addition to the risk of surgical wound contamination, these contaminants can also be inhaled by the occupants of the room and cause or worsen diseases [11,12,13,14]. In other words, the contaminants that are not removed from the air of an operating room, are also characterised as a potential risk of respiratory infection to the surgical team. For example, this can happen in orthopaedic surgery due to the smoke and aerosols produced by tools such as electrosurgical apparatus (bone saws, drills, etc.), which can remain in the air for long periods [15]. Such aerosols could also potentially be infected with pathogens and be spread all over the operating room, contaminating the animate and inanimate environmental surfaces [16]. The surgical personnel and environment are therefore exposed to these agents [17]. In addition, contaminants such as waste anaesthetic gases and CO_2_, if not controlled properly, can cause severe complications [18].

For these reasons, filtration and air exchange with outdoor air represent a key aspect in maintaining a controlled atmosphere inside operating rooms. Indoor particle concentrations are decreased by exhausting and diluting the return air with outdoor air; this process is referred to as dilution ventilation [19]. Dilution ventilation requires the system to have the capacity for conditioning the outdoor air before it is introduced into the room. With this renewal of air, a significant decrease in pollutants generated in the room or a reduction in concentration to a more acceptable and safe level can be obtained. That is, environmental air is mixed uniformly with outside air, facilitating dilution of contaminants inside the room. However, the outside air always contains contaminants such as bacteria, pollen, insects, soot, ash, dust, etc., just as the return air may contain these or other elements of contamination. Thus, the filtering system becomes indispensable in defining the quality of air inside the surgical room and is also responsible for protecting the facility, in particular the central air handling and ductwork, against any adverse effects of these materials.

Unfortunately, since their low price, simple installation, operation and maintenance, window/wall type air-conditioning or split systems have largely been used in operating rooms in Brazil. Normally, these types of systems only recirculate the air inside the room without appropriate filtration and renovation with outdoor air. Additionally, this kind of equipment produces horizontal jet flow and a large amount of turbulence in the room in contrast with the conventional downward flow systems [18,20]. Moreover, due to the way that this equipment moves the air inside the room, the surgical wound, people and surgical equipment are not protected against contamination. In the same way, it interferes with the pressure of the surgical room, potentially allowing pathogenic agents to come through other sections [21]. It is also important to highlight that, although window/wall air-conditioning and split systems are often used, there are few published studies that examine the impact of these types of equipment on indoor concentration and distribution of contaminants in operating rooms [20,21].

Generally, the level of CO_2_ generated indoors can be considered as good indicator of the efficiency of the air conditioning system in the process of air renewal [22]. In other words, elevated CO_2_ concentrations can be related to an increase of other indoor contaminant concentrations, which result from reduced ventilation [18,22,23,24]. CO_2_, being a common gas and a basic indicator of air quality, can also be used to simulate any other waste anaesthetic gas in operating rooms, since, normally, the CO_2_ contamination trend follows that of anaesthetics [18,20,22]. Additionally, CO_2_ concentration can be an indirect indicator of the occurrence of post-operative wound infections [18].

The CO_2_ concentration in the outdoor atmosphere is about 350 ppm, while indoors, a concentration of 700 ppm above the outside air can be regarded as an indication of the indoor air quality [23]. CO_2_ is a metabolic gas expelled, naturally, as a human respirational sub product. In order to define strategies for CO_2_ concentration measurement in confined environments, it is extremely important to define the variation in number of occupants, human activity level and ventilation rate.

Although window/wall air-conditioning and split systems are used all over the world in small surgery centres, few published studies have examined the impact of this type of equipment on particle and CO_2_ concentration levels. In this context, the objective of the present work is to analyse the performance of the window/wall air conditioning systems on indoor air ventilation in operating rooms with regards to CO_2_ and particle concentrations, since the latter can represent a vehicle for contaminant agents. To this end, CO_2_ and particle concentration levels were measured in a single surgical room conditioned with a window/wall type air-conditioning system, in a hospital specialised in treatment of infectious diseases, for two cases: single surgery and two subsequent surgeries in the same room.

## 2. Materials and Methods

### 2.1. CO_2_ Concentration and Ventilation in Buildings

Some research has confirmed that the measurement and the analysis of indoor CO_2_ concentration could be useful for understanding air quality and ventilation [25,26]. Indoor CO_2_ concentration is directly proportional to the number of people in a building and the ability of the ventilation system to dilute the CO_2_ generated from occupants.

The ASHRAE standard 62-2019 [25] estimates ventilation requirements based on CO_2_ generation as follows:

(1) $$Q=G/\left(C_{i}-C_{o}\right)$$

where *G* is the CO_2_ generation rate per person, *C_i_* and *C_o_* are the CO_2_ concentrations indoors and outdoors, respectively, and *Q* is the outdoor airflow rate per person. If the generation rate and the outdoor airflow rate per person are expressed in l/s, and the concentrations are in mg/m^3^, then the ventilation rate can be expressed as follows:

(2) $$Q=1.8\times 10^{6}\cdot G/\left(C_{i}-C_{o}\right)$$

Human metabolism consumes oxygen ($V_{O_{2}}$) and generates CO_2_ ($V_{CO_{2}}$) at rates that depend on the level of physical activity, body size and diet. Thus, the airflow rate per person can be expressed in terms of $V_{CO_{2}}$ as follows:

(3) $$Q=1.8\times 10^{6}\cdot V_{CO_{2}}/\left(C_{i}-C_{o}\right)$$

The CO_2_ generation rate of an individual is equal to $V_{O_{2}}$ multiplied by the respiratory quotient (*RQ*). The respiratory quotient, is the ratio of the volumetric rate at which CO_2_ is produced to the rate at which oxygen is consumed. The CO_2_ generation rate is 0.83 for an average adult engaged in light or sedentary activities increasing to a value of about 1 for heavy physical activity [26]. Thus, the generation of CO_2_ ($V_{CO_{2}}$) in l/s of a person is given by:

(4) $$V_{CO_{2}}=RQ\cdot V_{O_{2}}$$

The rate of oxygen consumption in l/s of a person is expressed as [3]:

(5) $$V_{O_{2}}=\frac{0.00276\cdot A_{D}\cdot M}{\left(0.23\cdot RQ+0.77\right)}$$

where *A_D_* is the DuBois body surface area (m^2^), *RQ* is the respiration quotient (ratio of CO_2_ exhaled to O_2_ inhaled) and *M* is the metabolic rate per unit of surface area (MET, $1MET=58.2\;W/m^{2}$). The DuBois surface area of a nude body can be estimated based on the body height and mas as reported in [3]. For a 20–50 years-old adult, the DuBois surface area ranges from 1.84 to 2.15 m^2^. The metabolic rate *M* is the energy dissipated by the body per unit of surface area to perform external functions (e.g., physical work, sports and daily tasks). Table 1 shows typical MET levels for a variety of activities for an average adult ($A_{D}=1.8\;m^{2}$) for activities performed continuously.

### 2.2. Measurement Localisation

**Figure 1:**
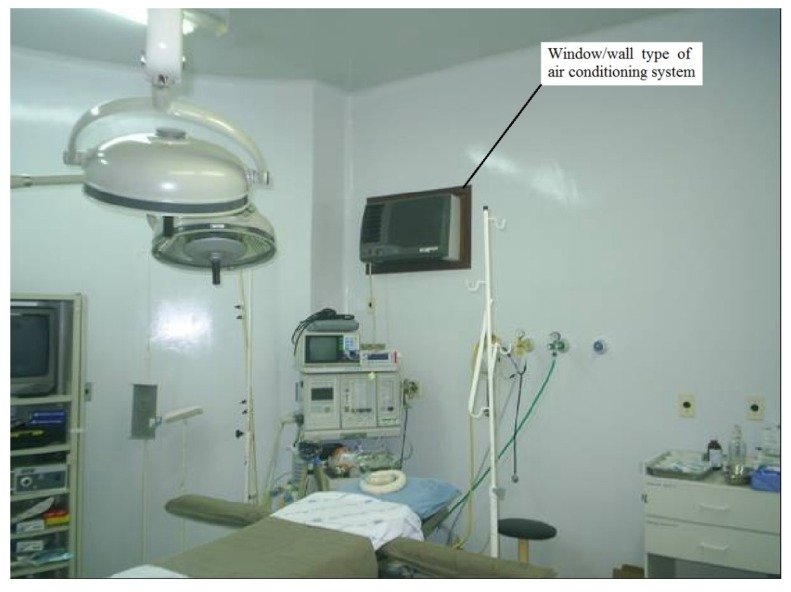
Surgical room and window/wall air conditioning system in which the CO_2_ and particle concentrations were measured.

The measurements were carried out in an operating room with an area of approximately 28 m^2^, in an old hospital for treatment of infectious diseases. The surgical centre of that hospital has just one surgical room. This surgical room has a window/wall type air conditioning system with a capacity of 5.3 kW. This type of equipment does not facilitate air renewal, and all components of the refrigeration cycle are in a single box. The indoor air enters through the side of the equipment, and is conditioned in its interior up to a certain temperature and humidity. It is then supplied to the indoor environment through the opposite side, by means of a fan. Figure 1 shows the surgical room with the window/wall air conditioning system in which the measurements were taken.

The measurements were carried out during different surgeries at a point chosen in the room as well as in the adjacent areas (corridor). Additionally, a comparison was made between the outdoor air requirements for ventilation necessary to maintain the indoor CO_2_ concentration within acceptable levels and the actual outdoor air ventilation provided by the ventilation system.

### 2.3. Instrumentation and Measured Parameters

It is important to highlight that none of the measurements carried interfered in the routine of the surgical procedure and were obtained during normal day-to-day routine in an operating room in Brazil. This study examined CO_2_ and particle concentrations during similar operations, performed in the same operating room and involving the same number of people. Measurements were taken simultaneously in the surgical room and in the corridor, starting before the surgery, with the empty room, until the patient left the room.

The particle concentration measurements were taken using two 6-channel Met One HHPC 6+ counters, calibrated by the manufacturer with a flow rate of 2.83 l/min, able to measure particle number concentration in the following range: 0.3 to 0.5 µm, 0.5 to 1.0 µm, 1.0 to 3.0 µm, 3 to 5 µm and 5 to 10 µm. Since the aim of the present paper is to evaluate the performances of the window/wall air conditioning systems by analysing the CO_2_ and particle concentrations before, during and after surgeries, the results in terms of particles are reported as the sum of the concentration measured for all the six channels (0.3 to 10.0 µm), which represent the size range of typical airborne infectious particles in operating rooms as described in the introduction section. Different measurements were made with an interval between each collection of 5 min and a sampling time of 1 min. Inside the room, during the whole measuring process, the measurement equipment was fixed onto the surgical light, above the patient at a distance of approximately 1 m from the surgical area.

The CO_2_ concentration levels were obtained through direct monitoring in real time. During the measurements the sensors were positioned away from any source that could directly influence the measure. A CO_2_ concentration meter (AZ Instrument Corp. model 77535 AZ, Taichung City, Taiwan) with a non-dispersive infrared sensor, feeding 20–30 V direct current, and with an analogue output of 4–20 mA was used to determine CO_2_ concentration. The apparatus was calibrated by the manufacturers and had a response time <60 s, accuracy ± 50 ppm and resolution ± 1 ppm.

The particle and CO_2_ concentration measurements were simultaneously carried out inside the surgical room and in the corridor adjacent to the room itself. For each surgery analysed, the measurements and recording of activities began before the patient entered the room, right after the cleaning of the room while it was still empty, and finished soon after the departure of the patient. The activities performed in the operating room were also recorded at 5-min intervals throughout the surgery to investigate the relationship between particles generated and activities performed in the room.

### 2.4. Estimates of the Outdoor Air Ventilation Requirements

In this study, a comparison was made between the outdoor air requirements for ventilation necessary to maintain indoor CO_2_ concentration within acceptable levels (700 ppm above outdoor air), and the actual outdoor air ventilation provided by the window/wall air conditioning system. The outdoor air ventilation requirements based on CO_2_ generation was determined using Equation (3) and considering as indoor CO_2_ concentration the actual measured value inside the operating room, while the required ventilation in order to maintain acceptable CO_2_ levels, was calculated considering an indoor CO_2_ concentration (C_i_) of 700 ppm above the outside air C_o_ (considering $C_{o}=350\;ppm$). Table 2 presents the values considered for calculation of the rate of CO_2_ generated per person, V_CO2_.

### 2.5. Statistical Analyses

To analyse the experimental data, SPSS Statistics version 16.0 from IBM (Armonk, NY, USA) was used to conduct descriptive statistical analysis and mean comparison. The Mann–Whitney test was used to compare the particle and CO_2_ concentrations between the cases analysed. The tests were considered significant at *p* < 0.01.

## 3. Results

Table 3 presents summary statistics for the concentration of the particle and CO_2_ measured inside the room and in the corridor. The average of the particle concentration inside the room was 22.3 particle/cm^3^ and CO_2_ concentration was 2470 ppm.

Table 4 Shows summary statistics for outdoor air requirements in l/s necessary to maintain the indoor CO_2_ concentration within acceptable levels, and the actual outdoor air ventilation provided by the ventilation system, calculated inside the surgery room and corridor. Outdoor air required inside the surgery room was 167 l/s, while the actual ventilation was 51 l/s.

**Figure 2:**
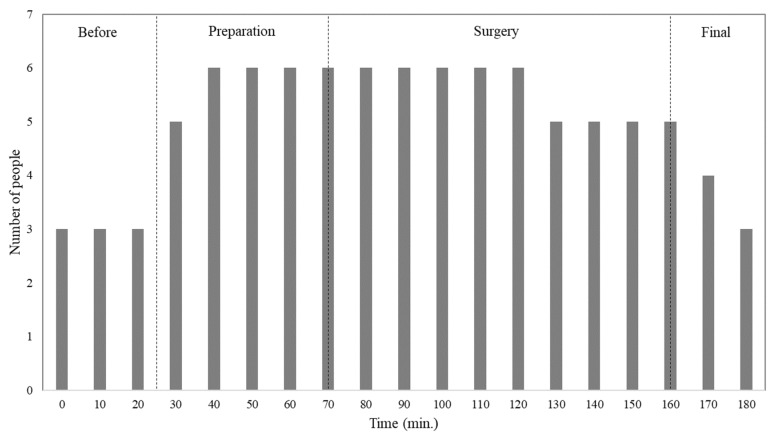
Number of people over time inside the surgical room, for both the cases of single surgery and two subsequent surgeries.

Figure 2 shows the variation in number of people in the room during the different surgical procedural steps, for both the analysed cases: single surgery and two subsequent surgeries. Initially, the number of people was three, which gradually increased during preparation time, arriving at six before the surgeries began. During the surgeries this value stayed at six for the majority of the time. Towards the end of the surgeries, the number of people fell to three.

Table 5 presents summary statistics for particle and CO_2_ concentrations within the operating room, for two subsequent surgeries (in the same surgical room). In the first surgery, particle concentration was 4.9 particle/cm^3^ and in the second was 29.3 particle/cm^3^. Data in Table 5 shows that particle concentration in the second surgery was approximately six times higher than in the first surgery (*p*< 0.01). In the case of CO_2_, the concentration in the first surgery was 1224 ppm and in the second was 1855 ppm. In other words, in the second surgery CO_2_ concentration was 1.5 higher than in the first surgery (*p*< 0.01).

**Figure 3:**
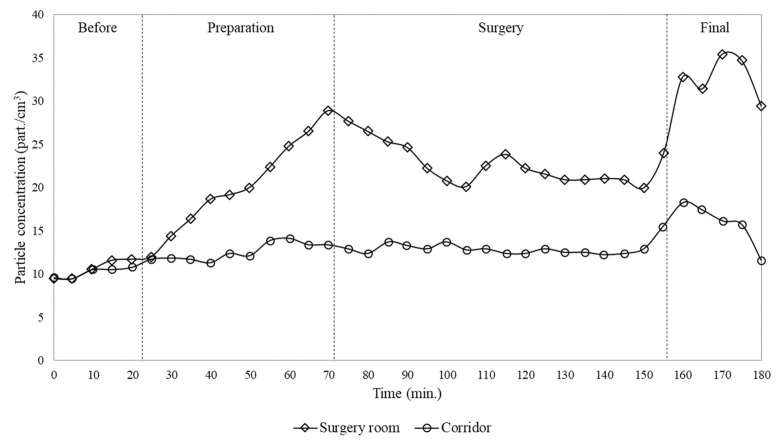
Time variation of particle number concentration (0.3–10 µm) measured inside the surgery room and in the corridor for the single surgery case.

**Figure 4:**
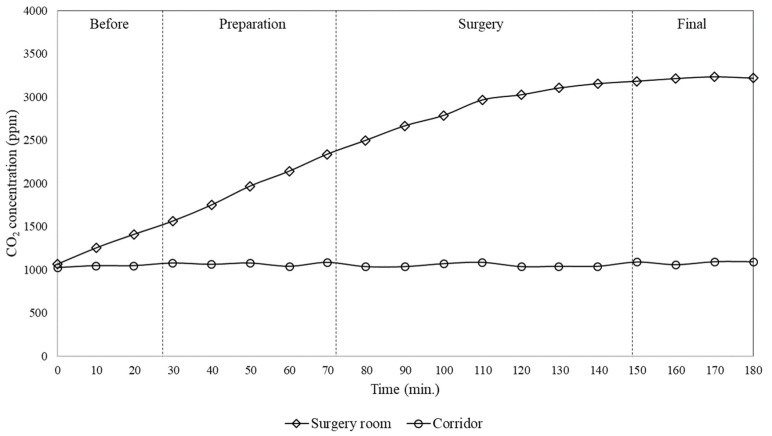
Time variation of CO_2_ concentration measured inside the surgery room and in the corridor for the single surgery case.

Figure 3 and Figure 4 show, for particle and CO_2_, a comparison between concentration in the operating room and in the corridor for the single surgery case. It was observed that before the patient entered the room, both particle concentrations were similar, at about 11 particles/cm^3^. When the level of activity inside the room became more intense, due to preparation for surgery, particle generation increased and consequently the particle level rose to values higher than in the corridor. By the end of surgery, particle concentration inside the room has increased up to 45 particles/cm^3^ while in the corridor has remained practically constant at around 12 particles/cm^3^.

With regard to CO_2_ concentration, it was observed that initially, before the patient entered the room, the CO_2_ level in both the room and corridor was at around 1000 ppm. This concentration inside the surgical room progressively increased during the start of activity reaching 3000 ppm by the end of the surgery, while during the whole procedure the level in the corridor practically remained constant, at approximately 1000 ppm.

**Figure 5:**
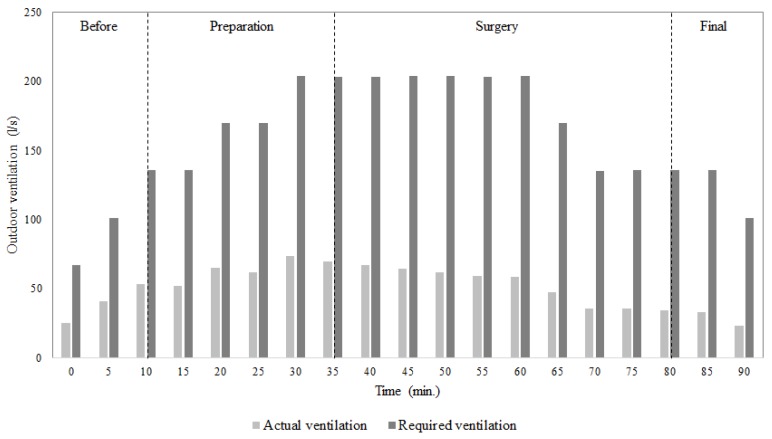
Comparison between the actual outdoor air ventilation provided by the ventilation system and the ventilation required to maintain acceptable CO_2_ concentration levels (700 ppm above outside air) inside the surgery room for the single surgery case.

Figure 5 shows a comparison between the outdoor air requirements for ventilation necessary to maintain the indoor CO_2_ concentration within acceptable levels, and the actual outdoor air ventilation provided by the ventilation system, for the single surgery case.

It can be seen that the outdoor air requirements were greater than the actual outdoor air ventilation provided by the ventilation system. This was observed especially at the end of preparation for surgery and for the surgery itself. In particular, during the surgery the outdoor air requirements were 200 l/s, while the real outdoor air ventilation provided by the ventilation system was only 70 l/s. These values were almost three times lower than outdoor ventilation necessary to maintain good indoor air quality. It can also be noticed that even before the patient entrance in the surgery room, when the number of people inside was small, the outdoor air ventilation provided by the system was not able to obtain acceptable indoor air quality.

**Figure 6:**
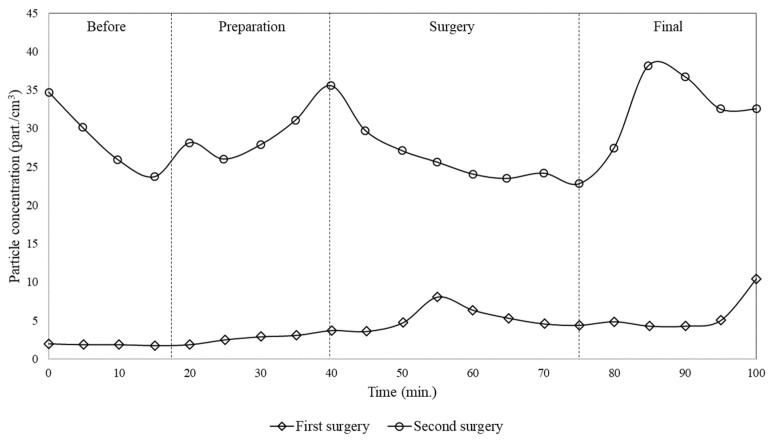
Time variation of particle number concentration (0.3–10µm) measured in the surgery room for the case of two subsequent surgeries.

**Figure 7:**
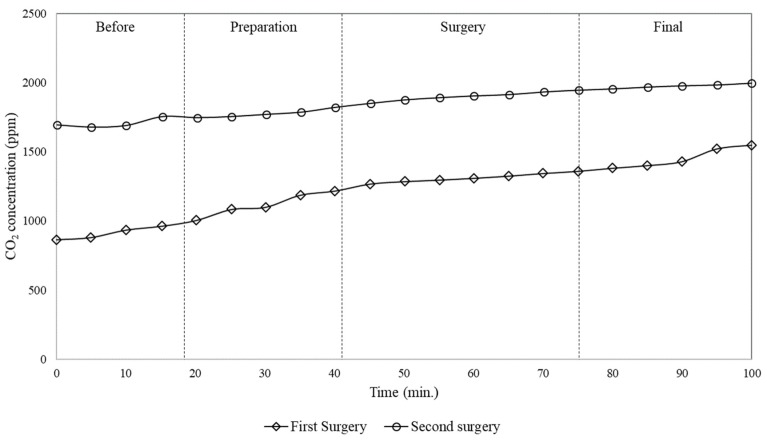
Time variation of CO_2_ concentration measured in the surgery room for the case of two subsequent surgeries.

Figure 6 and Figure 7 show comparisons between the concentration of particles and CO_2_ within the operating room, for the case of two subsequent surgeries. It was observed that the variation of particle and CO_2_ concentration during the first surgery was lower than during the second. It is important to highlight that between the two surgeries the room was cleaned for approximately 30 min, during which particles were re-suspended and/or generated, and additional CO_2_ was produced as well. Therefore, the difference between the concentration of particles during the two surgeries is not only due to the generation during the previous surgery but also to the cleaning process. Looking at Figure 6, it can be seen that during the second surgery the average particle concentration was about six times higher than during the first.

Figure 7 shows that the CO_2_ concentration measured during the second surgery was higher than during the first. In the first surgery, the concentration started at about 850 ppm and finished at above 1600 ppm. However, in the second surgery, the concentration was above acceptable values (about 1000 ppm) even before the patient entered. In the second surgery, the concentration started at approximately 1700 ppm and rose to around 2000 ppm.

## 4. Discussions

From the data reported above, it is possible to highlight typical behaviour for the time variation of particle and CO_2_ concentrations, which are in agreement with the results observed in other similar operating rooms during orthopaedic surgeries [17]. As regards the particles, before the patient entered in the surgical room, their concentration resulted lower if compared to other stages of the surgical procedure. Therefore, with the entrance of the patient and surgical team, and with the subsequent preparation for the surgical procedure, there was an increase in particle concentration due to intensified activities: movement of people, induction of anaesthesia, dressing with garments and drapes, etc. With the beginning of the surgery, the level of activity inside the room decreased and thus the generation and re-suspension of particles were less intense, resulting in a decrease in their concentration. At the end of the surgery, the level of particle concentration increased again due to movement of people.

In particular, from the measurements during surgeries a continuously increase in both CO_2_ and particle concentrations in the surgical room was observed, while the concentrations measured at the same time in the corridor remained practically constant. In addition, it was found that the outdoor air required in order to maintain the CO_2_ concentration below acceptable values (about 1000 ppm) was 167 l/s, while the actual installed window/wall air conditioning system provided only 51 l/s. In addition, particle and CO_2_ concentrations were measured within the operating room, for two subsequent surgeries, finding that the particle and CO_2_ concentrations in the second surgery were approximately 6 and 1.5 times higher than during the first, respectively. Furthermore, subsequent surgeries lead to a continuously increase of CO_2_ concentration and then of contamination level in the room, due to the activities of cleaning process and of previous surgeries. This behaviour was due to the characteristics of the window/wall air conditioning system installed, which was not able to provide the renewal of the air inside the room with outdoor air, but just its recirculation.

As already reported, there is a relationship between the CO_2_ production and other contaminants generated within an operating room, in particular with regards to anaesthetic gases. According to Spagnoli et al. [22], the CO_2_ concentration normally follows the same tendency as other gases such as anaesthetic gases. Thus, the results suggest that the concentration of anaesthetic gases inside the room would tend to be high during the surgical procedure. It is also important to note that exposure to high concentrations of anaesthetic gases, even for a short time, can cause adverse health effects. Some studies, in fact, have linked genetic damage of the surgical team with the exposure to anaesthetic gases in operating rooms [27]. Furthermore, high CO_2_ concentration levels, as found in this work, suggest that inadequate ventilation may lead to higher risk of surgical site infections due to high concentrations and long residence time of airborne contaminants in the surgery room.

The time variation of the particle concentration showed a reduction during the surgery, demonstrating that the flow pattern established by window/wall air conditioning systems can lead to significant particle deposition and consequently to surface contamination, since the analysed air conditioning equipment does not provide air filtration and renewal.

It also could be noticed that particle concentrations did not follow the same tendency of CO_2_ because they have different dynamics and deposition rates. CO_2_ is a gaseous pollutant that tends to diffuse in the air while particles, especially with the sizes analysed in the present work, tend to be deposited on the surfaces. This is a key aspect because considering that the analysed air conditioning equipment does not provide air filtration, the reduction of particle concentration, as observed in Figure 3, demonstrates that the flow pattern established by window/wall air conditioning systems can lead to significant particle deposition and consequently to contamination of instruments, people and surgical wounds.

Looking at Figure 6 and Figure 7, it can be stated that subsequent surgeries can lead to a continuously increase of CO_2_ concentration and then of contamination level in the surgery room, due to the activities of cleaning process and of previous surgeries. Thus, this suggests that if air conditioning systems does not provide adequate air filtration and renewal, as in the case of the window/wall air conditioning system, the air inside the surgery room can remain contaminated for long time.

## 5. Conclusions

In this study, the performance of the window/wall air conditioning systems on indoor air ventilation in operating rooms was analysed by measuring CO_2_ and particle concentrations inside the room and in the adjacent areas, for two cases of single surgery and two subsequent surgeries. The measurements and records of surgery activities began before the patient entered the room, right after the room was cleaned, and finished soon after the exit of the patient. Additionally, a comparison was made between the outdoor air requirements for ventilation necessary to maintain the indoor CO_2_ concentration within acceptable levels and the actual outdoor air ventilation provided by the ventilation system.

From the reported data, it can be concluded that the lack of air filtration and renovation with outdoor air in the surgical room is worrying, especially given that in these places the level of different contaminants generated can be quite high, putting the occupants in the risk of serious health concerns, a risk that may become even greater in case of surgical centres treating infectious diseases. Since the window/wall type air-conditioning or split systems are still commonly used in operating rooms in Brazil and many other countries, the results showed in this paper can be intended with a broad meaning, referring to all the cases in which the operating theatres are not provided with more sophisticated air conditioning system, specifically designed to filter and renew the indoor air and then protect the occupants against pathogenic agents.

It is important to highlight that in this work the measurements were carried out using an optical particle counter. This technology has some limitations, such as the possibility of interferences due to coincidence losses in particle counts at high particle concentrations. In addition, even if the outdoor air has a negligible influence on the indoor air quality since the analysed window/wall air conditioning system does not provide the renovation of indoor air from outdoors, the lack of data from parallel measurements conducted outside represent an aspect to take into account and that can have influence on the reported results. In the light of that, future work is needed especially considering parallel CO_2_ and particle concentration measurements on outdoor air, and comparing window/wall air conditioning system with other types of equipment and considering different operating theatres.

## Figures and Tables

**Table 1:** Typical MET (metabolic rate per unit of surface area) values for different activities.

Activity	MET
Seated, quiet	1.0
Reading and writing, seated	1.0
Typing	1.1
Filling, seated	1.2
Filling, standing	1.4
Walking at 0.89 m/s	2.0
House cleaning	2.0–3.4
Exercise	3.0–4.0

**Table 2:** Parameters for the calculation of the CO_2_ generated per person, V_CO2._

Parameter	Value
*A_D_* (m^2^)	2
*RQ*	1
*M* (MET)	2328

**Table 3:** Statistics for the concentration of the particle and CO_2_ measured inside of the surgery room and in the corridor.

	Particle (particles/cm^3^) (0.3–10µm)		CO_2_ (ppm)	
	Surgery room	Corridor	Surgery room	Corridor
Min	9.4	9.4	1040	1025
Average	22.3	12.9	2470	1064
Max	38.1	18.1	3222	1100
S.D.	7.6	1.8	639	21

**Table 4:** Statistics for outdoor air requirements to maintain CO_2_ concentration within acceptable levels (700 ppm above outside air), and the real outdoor air ventilation provided by the ventilation system, inside the surgery room.

	Required Ventilation (l/s)	Actual Ventilation (l/s)
Min	102	22
Average	167	51
Max	205	82
S.D.	43	19

**Table 5:** Statistics for particle and CO_2_ concentrations within the operating room, for two subsequent surgeries.

	Particulate Matter (particles/cm^3^) (0.3–10µm)		CO_2_ (ppm)	
	First surgery	Second surgery	First surgery	Second surgery
Min	1.9	23.0	865	1684
Average	4.9	29.3	1224	1855
Max	11.6	38.1	1550	2000
S.D.	2.9	4.5	204	106
